# Bryozoan–cnidarian mutualism triggered a new strategy for greater resource exploitation as early as the Late Silurian

**DOI:** 10.1038/s41598-022-19955-2

**Published:** 2022-09-16

**Authors:** Mikołaj K. Zapalski, Olev Vinn, Ursula Toom, Andrej Ernst, Mark A. Wilson

**Affiliations:** 1 University of Warsaw, Faculty of Geology, Żwirki i Wigury 93, 02-089 Warszawa, Poland; 2 Institute of Ecology and Earth Sciences, University of Tartu, Ravila 14A, 50411 Tartu, Estonia; 3 Department of Geology, Tallinn University of Technology, Ehitajate Tee 5, 19086 Tallinn, Estonia; 4 Institut für Geologie, Universität Hamburg, Bundesstr. 55, 20146 Hamburg, Germany; 5 Department of Earth Sciences, The College of Wooster, Wooster, OH 44691 USA

**Keywords:** Palaeoecology, Palaeontology, Ecology

## Abstract

Bryozoans were common benthic invertebrates in the Silurian seas. The large biodiversity among Silurian benthic organisms prompted diversified interactions, and as a result bryozoans hosted many other organisms as symbionts. Here we analyse the cystoporate bryozoan *Fistulipora przhidolensis* and unidentified trepostomes intergrown with auloporid tabulate corals and putative hydrozoans. The material comes from the uppermost Přídolí Series (Late Silurian) of the Sõrve Peninsula, Saaremaa, Estonia. Our analysis shows that the interaction was beneficial for both organisms—cnidarians benefited from feeding currents created by the host bryozoan, while the latter benefited from the protection from predators by cnidae, it can thus be classified as mutualism. Such associations are common in modern seas. The analysed organisms are typically encrusting when the symbiosis is absent, when intergrown they display erect, branching morphologies, raised over the substratum, thus exploiting a higher suspension-feeding tier. While similar associations were known from the Devonian, we demonstrate that this novel ecological strategy for greater resource exploitation started as early as the latest Silurian.

## Introduction

Bryozoans were among the most common Silurian benthic organisms. A large number of diverse bryozoans have been described from the tropical shelves of the palaeocontinent Baltica^1–5^. Besides bryozoans, large numbers (both in terms of diversity and biomass) of other organisms competed for seafloor space in tropical Silurian seas. Such competition prompted interactions between benthic organisms that resulted in a diverse network of interactions^6,7^. For example. representatives of the common cystoporate bryozoan genus *Fistulipora* have been observed to host diversified symbionts, such as rugose corals, cornulitids and others^8,9^.

Substrate space is an important and limited resource for benthic organisms^10^. Population size, survival and reproductive success are correlated with the area occupied by given organisms^11^. While diverse benthic species may use aggressive chemicals or toxins to repel potential predators or competitors^12^, the abilities of bryozoans to produce repellent chemical agents are probably rare^13^, and therefore these organisms are generally prone to overgrowths by other organisms. As a result, bryozoan–cnidarian associations are common in modern seas^14,15^, and numerous cases have been described from the fossil record ^16–18^. Among these, representatives of Hydrozoa are particularly common symbionts of bryozoans. In general, such hydrozoan-bryozoan associations are mutualistic^11^, where the bryozoan receives protection from the hydrozoan cnidae, while the hydrozoan profits from the feeding currents generated by the bryozoan^14^. Moreover, it has been shown that association with other organisms (in this case, other species of bryozoans) may influence the feeding current strength, and neighbouring colonies can profit from each other’s presence^19^, thus such an association may be desirable for both organisms involved. This can likely be extended to other bryozoan–cnidarian associations. While several such associations are known from the Devonian e.g.,^16,18,20,21^ they are less frequent in older strata e.g.,^9,22^.

It has been demonstrated that the association of two taxa can create a new ecological niche unavailable for each of the organisms separately. An instructive case was described by McKinney et al.^16^, who detailed mutualism between the tabulate coral *Aulopora* sp. and the trepostome bryozoan *Leioclema* sp. from the Lower Devonian of Tennessee, USA. While both taxa were generally encrusting, their intergrowth resulted in branching colonies, which enabled greater penetration of the water column for each organism than would have been available without the intergrowth. It therefore created a new ecological niche and allowed partial escape from the limited bottom surface. A similar case was described by Suárez-Andrés et al.^18,21^ from the Lower Devonian of Spain, who interpreted the relationship as commensalism.

**Figure 1:**
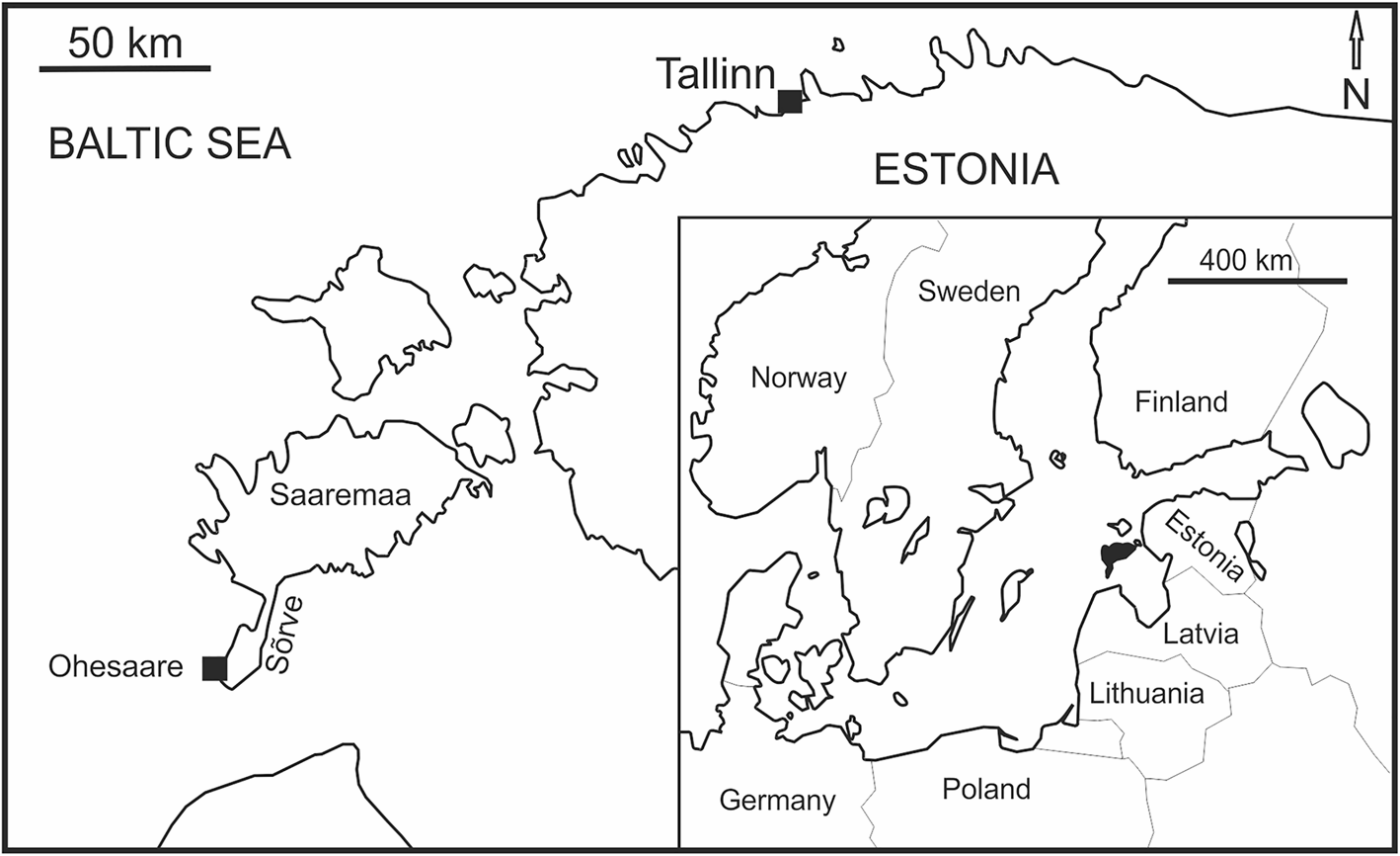
Location of the Ohesaare cliff on the map of Estonia and Europe. Based upon Vinn et al.^9^.

The aim of this paper is to describe and analyse examples of *Fistulipora przhidolensis* from the latest Přídolí Epoch exposed along Ohesaare cliff, Sõrve Peninsula, Saaremaa, Estonia (Fig. 1), which are intergrown with modular organisms of cnidarian affinities. Our material shows remarkable similarities to the Lower Devonian cases outlined above but is older by at least 5–7 Ma, thus pushing the appearance of such symbioses and the new niche deeper in time. While part of this material was briefly mentioned and illustrated by Vinn et al.^9^, it has not been separately analysed until now. In addition, we analyse two specimens of trepostome bryozoans from the same beds, hosting modular endosymbionts.

## Geological setting and palaeogeographical context

**Figure 2:**
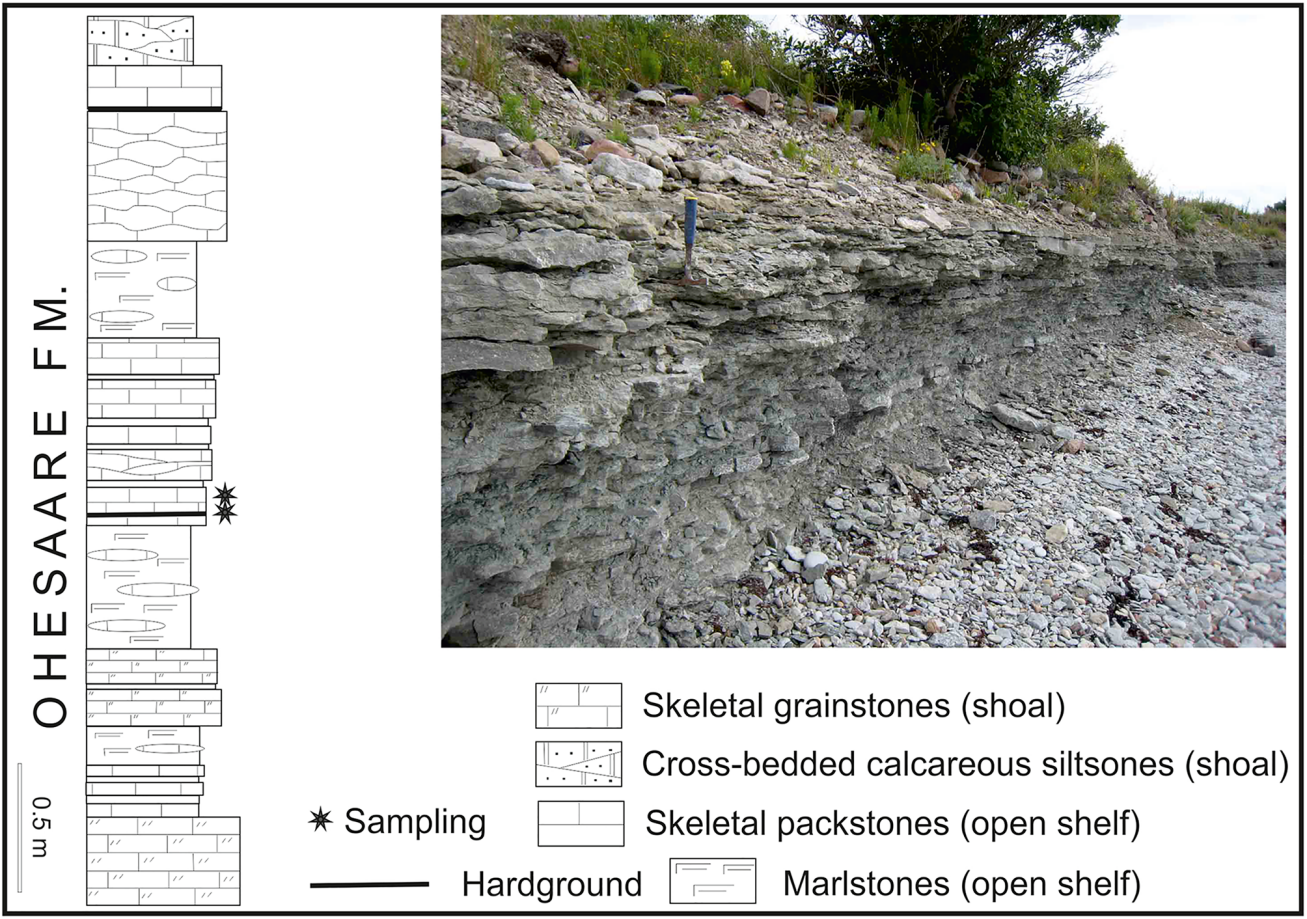
Detailed log of the Ohesaare cliff with indication of sampled bryozoan-bearing beds. Note the 28 cm long hammer for scale. Lithostratigraphic log taken from Vinn et al.^9^, based originally upon^28^.

During the Ohesaare age (latest Přídolí), the palaeocontinent of Baltica was at tropical latitudes, spanning from the Equator down to about 30°S^23,24^. A shallow epicontinental sea covered southernmost part of the today Saaremaa Island and its Sõrve Peninsula (Fig. 1). This shallow sea was characterized by tropical environments and diverse biotas^25^. Nestor and Einasto^26^ have described facies of the Baltic Silurian basin, including the Přídolí. They found five depositional environments in the Silurian of Estonia: tidal flat/lagoonal, shoal, open shelf, basin slope, and a basin depression. The first three environments formed a carbonate shelf, whereas sediments deposited in shoal and in open shelf environments are exposed on Saaremaa Island^27^. On the Sõrve Peninsula, the uppermost Přídolí strata (Ohesaare Formation) contain shallow to deeper shelf carbonate rocks, rich in shelly faunas. The best exposures of the Ohesaare Formation on Saaremaa Island are located on the west coast of the Sõrve Peninsula; the only uppermost Přídolí exposure is at the Ohesaare cliff (Fig. 1). The Ohesaare cliff is approximately 600 m long and has a maximum height of about 4 m (Fig. 2). The total thickness of the bedrock section is 3.5 m, whereas the thicknesses of individual beds are variable throughout the cliff^25^. The exposed rocks at Ohesaare cliff are typically an intercalation of thin-bedded limestones and marlstones^28^. The material used in this study originates from the clay-rich beds that are exposed at the base of the cliff, from the modern sea floor, and from skeletal packstones exposed directly above the lower hardground (Fig. 2).

## Results

The specimens represent small fragments of bryozoan colonies, usually not exceeding 2–3 cm. These colonies are in most cases branching, rarely irregular fragments. There are two groups of endobionts of possible cnidarian origin.

### Auloporid endobionts

**Figure 3:**
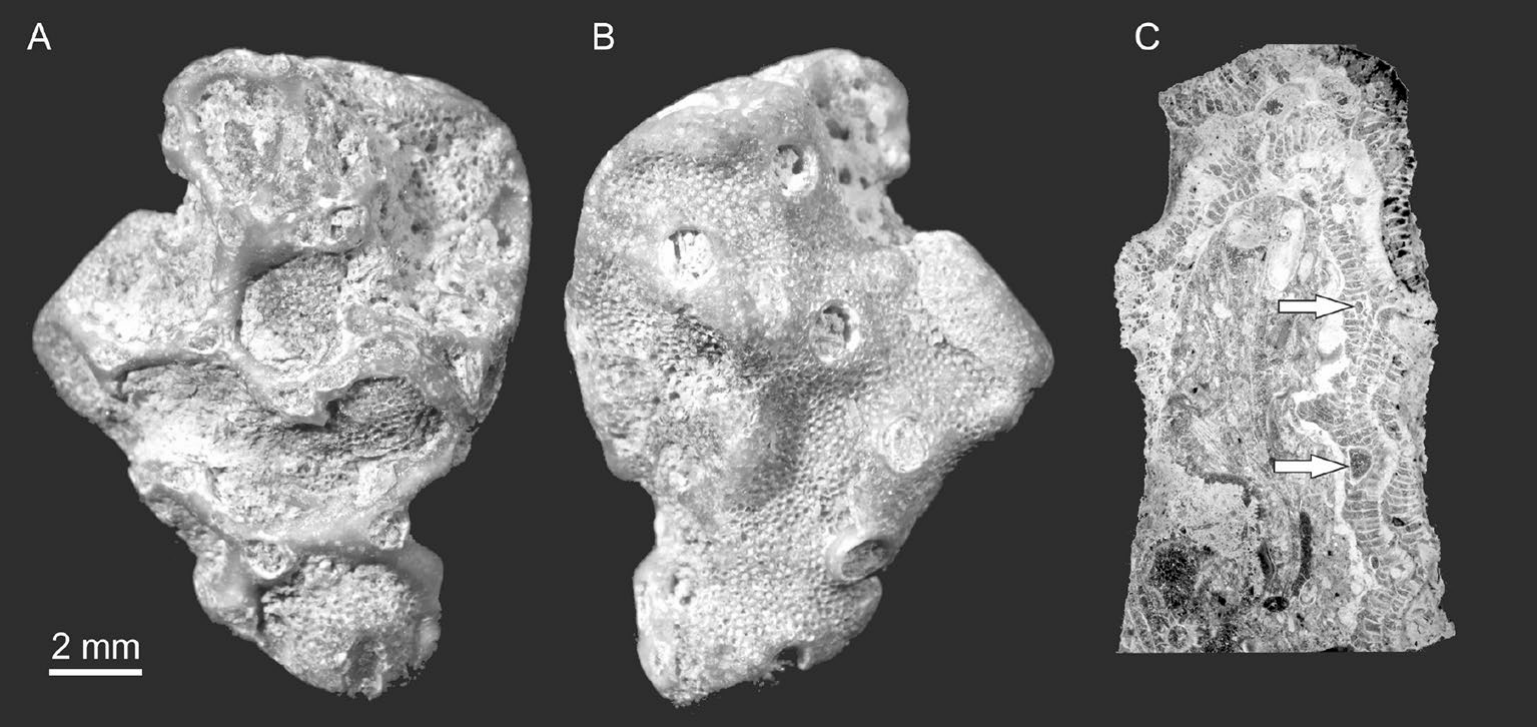
Auloporid corals (probably *A. amica*) intergrown with the bryozoan *F. przhidolensis*. Ohesaare cliff, Saaremaa, Estonia; Ohesaare Fm. Přídolí Series, Silurian. (**A,B**) Two sides of the same specimen. Note that the auloporid is partly encrusting the surface and partly embedded in the host bryozoan. Specimen GIT 403–419. (**C**) Thin section showing cross sections of auloporid corallites (arrow). Specimen GIT 403–261.

Three bryozoan colonies contain auloporids, most likely *Aulopora amica* (GIT 403–419, GIT 403–261, GIT 403–445^29^). One of the bryozoan colonies (GIT 403–419) contains an auloporid, visible on a small fragment as an encrusting colony on its surface; in its later astogenetic stages it was overgrown by the bryozoan. The diameters of the auloporid calyces in the free-living parts of colony are 0.8–1.3 mm; in the parts overgrown by the bryozoan these diameters reach 1.6 mm. A thin section (GIT 403–261) shows a similar situation where the auloporid was encrusting earlier growth stages of the host *F. przhidolensis* and was subsequently overgrown by the host colony (Fig. 3).

### Modular endobionts

**Figure 4:**
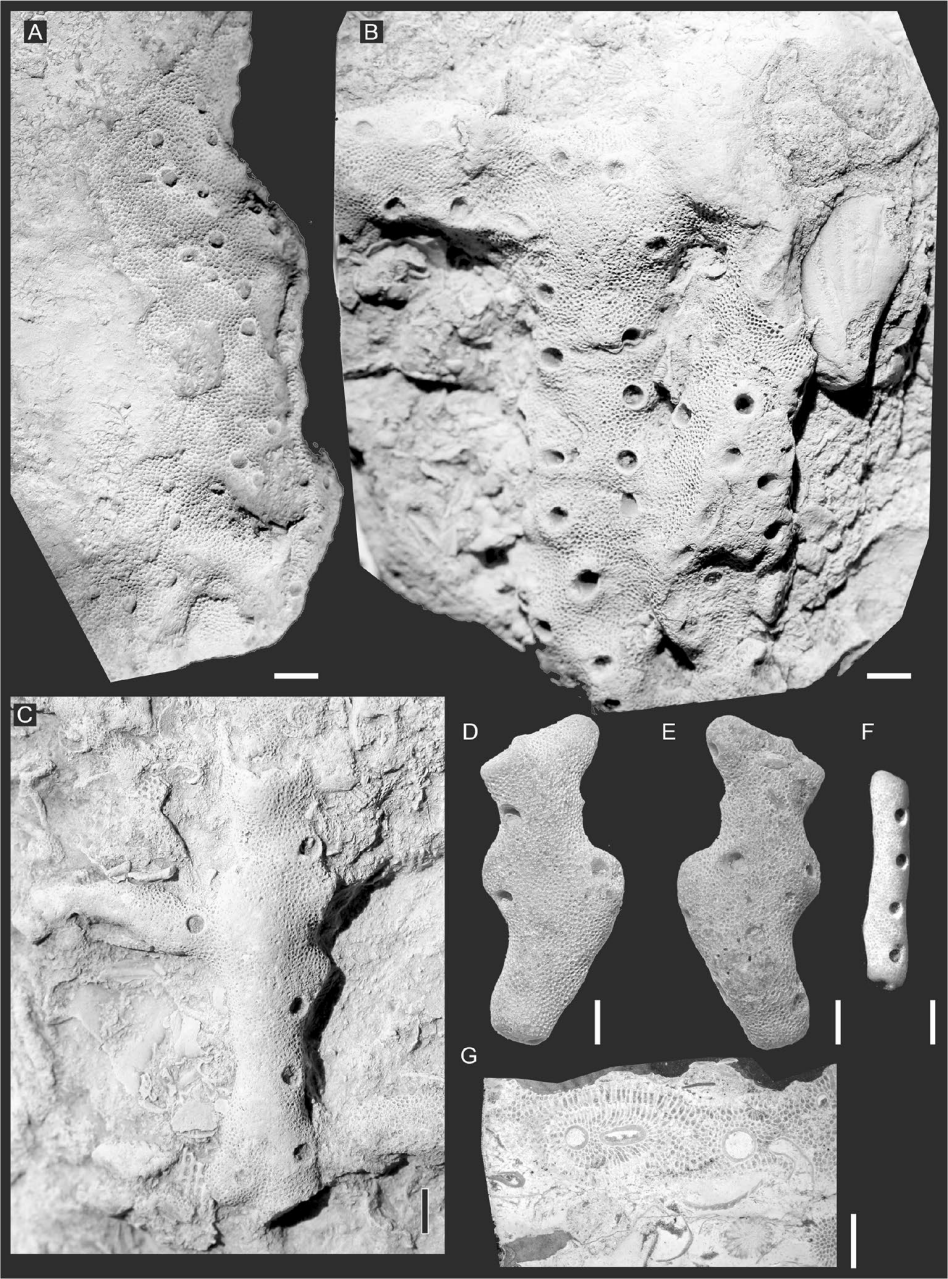
Modular endobionts, putative hydrozoans on *F. przidolensis* (**A–C,F,G**) and a trepostome bryozoan (**D,E**). Ohesaare cliff, Saaremaa, Estonia; Ohesaare Fm. Přídolí Series, Silurian. (**A**) Specimen GIT 403–261; (**B**) specimen GIT 403–416, figured by Vinn et al.^9^. (**C**) Specimen GIT 403–415. (**D,E**) two sides of the specimen GIT 403–420. (**F**) GIT 403–421, (**G**) thin section GIT 403–416. Scale bars 2 mm.

**Figure 5:**
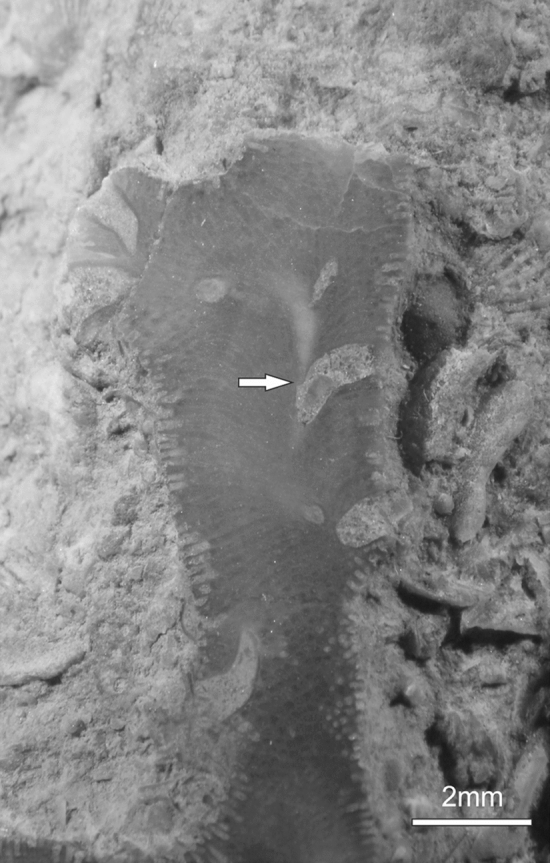
Modular endobionts, putative hydrozoans. Ohesaare cliff, Saaremaa, Estonia; Ohesaare Fm. Přídolí Series, Silurian. Polished slab. Note proximal budding (arrow). Specimen GIT 403–418, figured by Vinn et al.^9^.

Other specimens usually (*F. przhidolensis*: GIT 403–304; 403–415; 403–417; 403–418; 403–421; 403–422; 403–445; 403–451; 403–474; 403–634; 403–697; TUG 1743–135; Trepostomes: GIT 403–416; 403–420; Fig. 4) display usually regularly distributed holes, 0.6–0.9 mm in diameter and usually spaced 15–25 mm apart (measured as border to border), rather evenly distributed within a given host bryozoan colony. In one case, these holes form two, quite regular, parallel rows (specimen GIT 403–416, Fig. 4B). On a clearly branching specimen the openings are distributed along the axis, alternating from one side to the other, thus geniculate (e.g., GIT 403–421, Fig. 4F). In thin sections and slabs, the budding is visible, which proceeded at the base of the parent individual (Fig. 5). The structures possess their own walls of variable thickness, 0.012–0.016 mm, which is not significantly different from the thickness of the host’s walls. As shown by EDS analysis, their composition is carbonate (domination of Ca and locally Si) and does not show signs of phosphatization (entire lack of P). Thin section and specimen examination did not reveal any carbonization.

## Discussion

### The biological affinity of the endobionts

The endobionts of the first group clearly belong to auloporids, identified here by their similar morphometric characteristics to *Aulopora amica*^29^. Auloporids are classified within Anthozoa, subclass Tabulata^30,31^.

The affinities of the second group of specimens are more interesting. Such modular morphology suggests cnidarian, bryozoan or hemichordate affinities. The investigated organisms of the second group display very simple morphology. They are geniculate, with openings alternating in many cases on both sides of the branch. This pattern commonly occurs in the material but is not consistent; it might be possible that our material represents more than one taxon. Geniculate colony morphology with distal budding and funnel-shaped calyces was described from fossil *Cladochonus* organisms^30,32,33^. This genus is characterized by funnel-shaped “calices”, uneven walls and lack of internal structures, such as tabulae or pores^33^. Our material shows strong resemblance to *Cladochonus*. Its taxonomic position has been discussed for a long time^32^, and it was traditionally assigned to Pyrgiidae within the subclass Tabulata^30^, but Stasińska^32^ pointed out deep anatomical differences between *Cladochonus* and tabulates on one hand, on the other she demonstrated its resemblance to modern hydrozoans. Król^33^ classified it as Incertae Sedis, while Coronado pointed microstructural features and concluded that *Cladochonus* is likely to be a calcifying hydrozoan^34^, a point of view we adopt here. It can be further supported by the fact that this kind of colony structure commonly occurs in various modern hydrozoans, such as representatives of Campanulariidae (e.g., *Obelia*) or Tiarannidae (e.g., *Stegolaria*)^35^. Apart from pyrgiids, such colony structure is unknown in both tabulate and rugose corals^30^.

The mode of preservation of our material does not show any signs of carbonization. While the budding pattern may suggest hemichordatan affinity^36^, the EDS analysis did not reveal any signs of phosphorus. Phosphorus presence could suggest chitinous remains^37^, and organic tubes are typical for hemichordates^38^, which are often preserved as carbonized remains^39,40^. Also, hemichordates, such as graptolites, have their thecae much smaller than in the material discussed here. Hemichordatan affinity therefore seems unlikely.

Also, bryozoan affinity can be ruled out, as the studied endobionts show no similarities to bryozoans. At first, the size of the modules (0.6–0.9 mm in diameter) exceeds the usual size of autozooecia in bryozoans. Their spacing is also too large for bryozoans (15–25 mm). Bryozoans are suspension feeders, therefore they need optimal distance between the tentacle crowns, so that tentacles can effectively operate in this space. The large spacing would assume presence of very long tentacles (more than 7–12 mm) which are unknown in bryozoans.

To sum up, it seems that our material resembles representatives of the Pyrgiidae family, notably *Cladochonus*. Following Stasińska^32^ and Coronado^34^, we accept its putative hydrozoan affinity, therefore our endobionts belong to Anthozoa (Auloporida) and Hydrozoa (?Pyrgiidae).

### The interaction between host bryozoan and the endobionts

The presence of the endobiont apparently does not cause positive or negative modifications of the host bryozoan. Its skeleton is modified in the sense that zooids surround the endobiont, but without other type of modification. Tapanila^41^ published a list of criteria to distinguish between various kinds of symbioses; skeletal modifications are needed in order to choose from any of those. In cases of lacking modifications, Tapanila^41^ proposed commensalism as a null hypothesis in palaeoecology. While commensalism is often reported in both recent and fossil communities (see reviews^42,43^) it has been shown that detecting commensalism is unlikely in the fossil record^42^ due to lack of evidence of the lack of interaction. According to Mathis and Bronstein^43^, many studies on recent interactions have shown that evidence of any kind of interaction is truly absent. As a result, it is likely that it occurs in modern associations, despite lack of evidence.

While commensalism cannot be shown in this studied case, the nature of the relationship can be inferred from general knowledge of the biology of both organisms. While auloporids and pyrgiids were common parasites of echinoderms in the Palaeozoic^44^ it seems that this is not the case here. Bryozoans are suspension feeders^45–48^. They can create feeding currents, and as a result food particles flow towards their colony when it is active^19,48^. Therefore, an organism associated with the bryozoan colony can benefit from feeding currents, either impoverishing the particle composition, or capturing particles too large for the host bryozoan to swallow and digest. On the other hand, an endobiont, assuming that it is cnidarian, can protect the host with its cnidae^14^. As such an interaction is beneficial for both involved organisms and can thus be classified as mutualism. Research on hydrozoan-bryozoan associations demonstrated that such host-symbiont interactions evolved independently in several groups of hydroids^14^, and such associations are relatively common in recent seas^49–51^.

Mutualistic interrelations between colonial (or modular) organisms are widespread because of their ecological plasticity and similar ecological needs^52^. As seen from the previous discussion, two main factors are considered in mutualistic interrelations between bryozoans and cnidarians: feeding and protection. Colonial animals may also undergo constructional modifications in the process of adapting to their substrates to achieve mutual benefit. Encrusting organisms face substantial problems on the substrate such as space and food limitations (e.g.,^53^). Bryozoans are poor competitors for space on the substrate against other animals^54,55^; space and food limitations on the substrate can be avoided by developing erect forms and thus achieving higher feeding tiers^52,56^.

An example of mutual intergrowth similar to that represented here was described by McKinney et al.^16^. The trepostome bryozoan *Leioclema* sp. and the coral *Aulopora* sp. from the Lower Devonian of USA produced erect constructions, with a coral inside branches of the host bryozoan colony. It is supposed that the bryozoan and the coral benefitted from this association, first to escape from the limited space on the substrate and second to obtain tiered space for feeding. The material described by McKinney^16^ comes from the Birdsong Shale Member of the Ross Formation, which is middle Lochkovian^57^. The example presented here shows that such an ecological innovation appeared as early as in the Late Silurian, therefore it is 5–7 million years older than previously described.

Similar interrelations can be proposed for the material from the Silurian of Saaremaa. The bryozoans involved in the symbiosis were normally encrusting species. The species *Fistulipora przhidolensis* often produced unilaminar encrusting or globular multilayered colonies. Due to intecactions with the auloporid coral and a hydrozoan, erect colonies appeared which allowed occupation of higher tiers for feeding. Such a strategy benefits both involved organisms and helps them limit substrate competition and to exploit new food resources higher in the water column. As shown in Recent examples, cnidarians can limit the number of predators on bryozoans whereas the latter protect hydroids by enveloping their soft parts with calcitic skeleton^11,14,49,58,59^. It can therefore be assumed that protection by cnidae also played an important role in this interaction. The surface of the coral/hydrozoan was covered by the encrusting bryozoan, whereas the bryozoan might be protected by action of the cnidae of the cnidarian tentacles. Moreover, the cnidarian can profit from the feeding currents produced by the bryozoan. In contrast to cnidarians, bryozoans are active suspension feeders which produce feeding currents due to orchestrated movement of cilia on their tentacles (e.g.,^60^ and references herein). Cnidarians are incapable of actively creating feeding currents; they only catch the prey within their reach. Bryozoans and cnidarians are not food competitors: the former feed on smaller phytoplankton, whereas the latter utilize the larger zooplankton^61^.

We can easily rule out the alternative explanation that the observed tubes are a result of borings in the host bryozoan skeletons. If that was the case the bryozoan zooecia would be cut by the boring randomly^62,63^ and would not encircle the endobiont as they do in our material (Fig. 4G).

### Bryozoan symbiotic endobionts in the Early Palaeozoic

Bryozoans are known to have formed symbiotic associations with other invertebrates since the Tremadoc^64^. Endobiotic invertebrates with phosphatic tubes and some with entirely soft bodies formed symbiotic associations with the trepostome *Orbiramus* in the Tremadoc of China^64^. These earliest bryozoan symbiotic endobionts were solitary animals with unknown biological affinities. However, phosphatic tubes are characteristics of the presumed cnidarian *Sphenothallus* known from the Tremadoc, though the tubes described by Ma et al.^64^ are slightly too small for *Sphenothallus*. Nevertheless, tubicolous morphology and *Sphenothallus*-like composition could indicate a cnidarian affinity.

The Great Ordovician Biodiversification Event resulted in appearance of dense ecological interactions in benthic communities and in consequence a number of new ecological niches, or ecospaces appeared^65^. As a result of biodiversity increase, and following increase of competition, Ordovician bryozoans often formed symbiotic associations with cnidarians such as solitary rugose corals and conulariids. These cnidarians were more common bryozoan symbionts than the suspension feeding cornulitid tubeworms^22,66^. While the latter were solitary forms, the earliest colonial bryozoan endobionts interpreted as hydroids or ascidiacian tunicates appeared in the early Late Ordovician of Laurentia^67^. It must be emphasized that the Ordovician record of bryozoan endobionts was dominated by solitary organisms. On the other hand, in the Přídolí, colonial animals became much more prevalent, with still significant contributions from rugose corals and *Cornulites*^8,9^. The abundance of colonial endobionts among the Přídolí bryozoans from Saaremaa could have resulted from locally favourable environmental conditions, faunal composition and lack of antifouling agents in bryozoans. Nevertheless, it is also possible that the importance of colonial bryozoan endobionts increased from the Ordovician to Silurian. It seems that cnidarians were dominant bryozoan endobionts in the Přídolí. One could hypothesize that cnidarian biology fit well with bryozoans as they likely consumed food particles of different sizes and cnidarian symbionts could protect the host bryozoan with its cnidae. Last, but not least, a successful mutualism, allowing the use of a new feeding tier could prompt its more common appearance, as evidenced by a number of similar associations described from various parts of the World—e.g., the Early Devonian of Spain^18^, and Czechia^68^ or Middle Devonian of Russia (Kuznetsk Basin^69^). For a review on palaeogeographical distribution of such forms, see^18^. The appearance of similar symbiosis in cystoporates and trepostomes also shows the success of the newly created niche.

## Conclusions

We have shown that during the Přídolí the bryozoan *Fistulipora przhidolensis* and unidentified trepostomes formed associations with two different representatives of cnidarians. Auloporid tabulate corals belong the first group, while the representatives of the other most probably belong to “*Cladochonus*-like” fossils, which were most likely hydrozoans. Both organisms (bryozoans and cnidarians) usually formed flat, encrusting colonies when growing separately; when intergrown, they formed branches. Such a modification between free-living and symbiotic mode of life shows the appearance of a new ecological niche for both involved organisms. While the skeletal modifications of the host bryozoans are absent, it can be inferred that the interaction between them was mutualistic, where cnidarians profited from the feeding currents generated by the host bryozoan, and the bryozoan benefited from the protection by the cnidarian cnidae. Such mutualistic associations are common in modern seas. This mutualism therefore introduced a structural innovation, where both organisms started to exploit a new, higher tier of suspension feeding unavailable for them separately. Such an association is known from several Devonian sites around the world, which demonstrates its ecological success,. Our study shows that this innovation appeared 5–7 Ma earlier than the oldest known example, in the Přídolí (Late Silurian).

## Material and methods

A collection of about 500 bryozoan colonies from Přídolí sediments of the Sõrve Peninsula, Saaremaa, Estonia (Ohesaare Formation), was searched for the intergrowth of different invertebrates. The present work analyses the material of 17 specimens, which contain 20 fragments of the bryozoan *Fistulipora przhidolensis* hosting modular bioclaustrations. The material comes from the Ohesaare cliff on Saaremaa, Estonia (Fig. 1). Five thin sections of selected specimens were prepared to investigate their internal structure. Specimens and thin sections were studied under a Zeiss Discovery.V20 stereoscopic microscope under reflected and transmitted light. The specimens were photographed using ammonium chloride coating with a Canon EOS 70D camera either using Zeiss Discovery.V20 stereoscopic microscope or using Canon EF 100 mm f/2.8L Macro IS USM Lens. Specimens photographed under the microscope were uncoated. Helicon software was used to stack photos of selected specimens in order to obtain the best depth-of-field. Thin sections were photographed using transmitted light and dark field. Selected specimens were also photographed using SEM—ZEISS AURIGA 60, Energy Dispersive Spectroscopy (EDS) analyses on one uncoated specimen was performed with Zeiss Sigma VP SEM at the Faculty of Geology, University of Warsaw. Brightness, contrast and sharpness of images was adjusted in Corel Photo Paint software, each time with the whole image.

## Data Availability

The investigated material that supports this study is available at the Natural History Museum of the University of Tartu (collection numbers with a prefix TUG) and Department of Geology of the Tallinn University of Technology (institutional abbreviation GIT).
